# Pattern-related profiles of acupuncture points for pain management: A retrospective study of outpatient data from Korean medicine clinics

**DOI:** 10.1097/MD.0000000000050804

**Published:** 2026-09-18

**Authors:** Da-Eun Yoon, Yoonjeong Seo, Heeyoung Moon, Shin Ho Kong, Changwoo Nam, Joowon Hwang, Karam Kim, Man-Heum Kwon, Hyun-Woo Jin, Yeonhee Ryu, In-Seon Lee, Younbyoung Chae

**Affiliations:** a Department of Science in Korean Medicine, Kyung Hee University, Seoul, Republic of Korea; b Department of Meridian Medical Science, Graduate School, Kyung Hee University, Seoul, Republic of Korea; c Department of Meridian and Acupoints, College of Korean Medicine, Semyung University, Jecheon, Republic of Korea; d Misodam Korean Medicine Clinic, Busan, Republic of Korea; e Haenamu Korean Medicine Clinic, Seongnam-si, Gyeonggi-do, Republic of Korea; f Chungpoong Korean Medicine Clinic, Seoul, Republic of Korea; g Kyunghee Ilsaeng Korean Medicine Clinic, Seoul, Republic of Korea; h H-Nuri Korean Medicine Clinic, Namyangju-si, Gyeonggi-do, Republic of Korea; i Cleanwood Korean Medicine Clinic, Incheon, Republic of Korea; j Korean Medicine Science Research Division, Korea Institute of Oriental Medicine, Daejeon, Republic of Korea.

**Keywords:** acupoint indication, hierarchical clustering, pain management, pattern identification, real-world data

## Abstract

In Traditional East Asian Medicine, acupoints are selected based on clinical symptoms and pattern identification. Since treatment plans target specific diseases and patterns, acupoints may be selected based on clinical indications or pattern-related considerations. This study aimed to examine the associations between acupoint use and deficiency–excess, cold–heat, and dryness–dampness pattern scores. Data were collected from the medical records of 423 outpatients with various pain disorders. Clinicians assigned semi-quantitative scores for deficiency–excess, cold–heat, and dryness–dampness on a −5 to +5 scale as part of the study assessment. Mean scores across the 3 pattern dimensions were calculated for the 30 most frequently prescribed acupoints, followed by hierarchical clustering. Two- to six-cluster solutions were compared using silhouette scores, and a five-cluster partition was retained for descriptive interpretation and further assessed for bootstrap stability. Across the 423 cases, ST36, LR3, and LI4 were the most commonly used acupoints for pain management. These 30 acupoints showed heterogeneous profiles across the 3 pattern dimensions. Hierarchical clustering provided an exploratory five-group representation, although no uniquely optimal cluster solution was identified. Commonly used acupoints showed heterogeneous profiles across clinician-assigned deficiency–excess, cold–heat, and dryness–dampness scores. These findings provide an exploratory description of pattern-related tendencies in real-world acupoint selection. Further prospective studies using standardized pattern assessment and adjustment for disease- and clinician-related factors are required to validate these associations.

## 1. Introduction

In Traditional East Asian Medicine (TEAM), acupuncture is preceded by a diagnostic process known as “pattern identification.”^[1,2]^ This process assesses the patient’s overall condition based on various signs, including complexion and pulse and classifies it into diagnostic patterns. Based on these diagnostic findings, personalized treatment plans are developed. Acupuncture points (acupoints) are used to address primary symptoms (branch treatment) or the underlying pattern of illness (root treatment).^[1]^ Given the large number of acupoints, examining their clinical use may help clarify how they are selected. The use of acupoints for particular diseases or patterns can be inferred from relationships between patient diagnoses and acupoint prescriptions.^[3]^ Acupoints may therefore be selected based on their clinical indications or their ability to address particular patterns.^[4]^ In this study, “clinical feature” refers to the pattern-related context in which an acupoint was prescribed, whereas “indication” refers to specific diseases or symptoms.

Acupoints are used to treat various types of pain.^[5,6]^ Clinical trials have demonstrated the effectiveness of acupoints for specific diseases and pain locations.^[7]^ Examining the relationship between pattern identification and acupoint selection poses a challenge because patterns are difficult to measure and standardize.^[8]^ Nevertheless, understanding how pattern identification relates to acupoint selection remains important in TEAM because pattern identification plays a central role in treatment planning.^[9,10]^ Examining these relationships may help characterise acupoint selection from a holistic perspective by clarifying how disease-specific indications and broader pattern-related considerations jointly shape in clinical decision. Previous studies have reported the association between pattern identification and prescribed acupoints in virtual diagnostic cases.^[11]^ However, these studies have not investigated how pattern identification relates to acupoint selection in real-world clinical practice.

In the current study, we conducted a retrospective network analysis of real-world clinical data from Korean Medicine clinics. We identified commonly used acupoints for pain management and described their associations with deficiency–excess, cold–heat, and dryness–dampness dimensions.

## 2. Methods

### 2.1. Data collection

Data were collected from the medical records of 423 outpatients treated at 7 Korean Medicine clinics between September 2022 and May 2023. The included conditions were low back pain, migraine, irritable bowel syndrome, ankle sprain, knee pain, carpal tunnel syndrome, and dysmenorrhea. Data included patient demographics, pain characteristics (intensity, duration, and pattern), acupoint prescriptions, and other interventions. Prior to data collection, clinicians were instructed to assign pattern scores based on their routine diagnostic process using a −5 to +5 scale, with 0 representing a clinically neutral or balanced state and increasing absolute values reflecting greater deviation toward either side of each pattern dimension. These scores were used as semi-quantitative representations of clinicians’ pattern assessments and were not intended as standardized diagnostic measurements. Each patient was assessed by a single treating clinician, and formal inter-rater reliability assessment or calibration across clinicians was not performed. Only data from initial visits were included to avoid potential changes in pain patterns due to disease progression. This study received exempt certification from the Institutional Review Board of Kyung Hee University (KHSIRB-23-065).

### 2.2. Acupoint combinations for pain control

We used VOSviewer software (version 1.6.19, https://vosviewer.com) to perform a network analysis of acupoint combinations for pain management. Network visualization was constructed using a co-occurrence-derived similarity matrix of acupoints.^[12]^ VOS mapping, a technique that quantitatively describes relationships between nodes representing individual items, was used to identify clusters.^[12]^ Acupoints appearing in more than ten of the 423 cases were included for analysis. The relative importance of each acupoint was determined based on its frequency of occurrence.

### 2.3. Analysis of common acupoints for pain control

**Figure 1.**
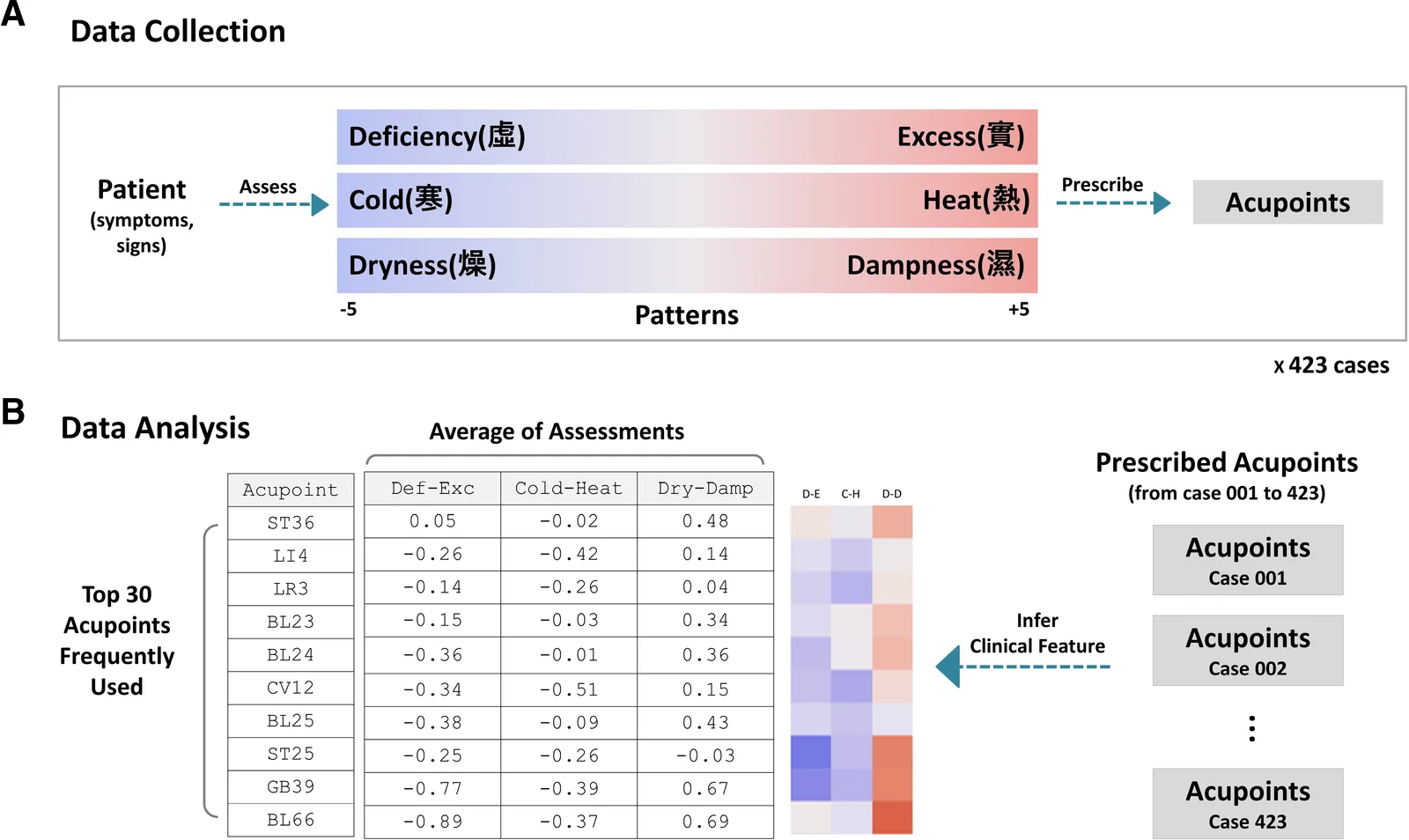
Study procedure and acupoint analysis. (A) Outpatient data from 7 clinics were collected for patients with pain disorders. Pattern assessments (deficiency-excess, cold-heat, dryness-dampness) were conducted on a numerical scale (−5 to +5), with lower values indicating deficiency, cold, or dryness. Prescribed acupoints were recorded. (B) Schematic illustration of the analytical procedure. Example mean pattern assessment values for frequently prescribed acupoints are displayed as a heatmap.

To analyze associations between acupoints and common pathological patterns, we focused on 3 fundamental imbalances: deficiency–excess, cold–heat, and dryness–dampness. These patterns represent holistic imbalances in the human body.^[13]^ Korean medical doctors assessed the severity of each pattern on a numerical scale ranging from −5 to +5, with lower values indicating a greater degree of deficiency, cold, or dryness (Fig. 1A).

To assess the relationship between acupoint use and pattern identification, we analyzed 3 pattern dimensions: deficiency–excess, cold–heat, and dryness–dampness. Based on 423 cases, we first identified the 30 most frequently prescribed acupoints through a frequency analysis. For these acupoints, mean deficiency–excess, cold–heat, and dryness–dampness scores were calculated and visualized in a heatmap (Fig. 1B). Mean scores were calculated for each acupoint based on the cases in which it was prescribed. These mean scores reflect the pattern profiles of patients for whom each acupoint was prescribed and do not represent intrinsic properties of the acupoint itself. Additionally, an analysis of the average pattern assessments across all cases was conducted to investigate the prevailing patterns observed in pain disorders.

### 2.4. Hierarchical clustering analysis of the acupoints

To group acupoints according to similarity in their associated pattern profiles, hierarchical clustering was performed. Using the numeric dataset of pattern assessments, the 30 selected acupoints were clustered. Hierarchical clustering, a widely used method for grouping data points based on a distance matrix, was applied.^[14]^ We used Euclidean distance without normalization and Ward minimum variance method to minimize within-cluster variance.^[15]^ To assess the clustering structure, silhouette scores were calculated for solutions ranging from 2 to 6 clusters.^[16]^ Because silhouette values were relatively similar across candidate solutions, the analysis was not considered to identify a uniquely optimal number of clusters. The five-cluster solution was retained as a higher-resolution descriptive representation of the pattern-related profiles based on the hierarchical structure and clinical interpretability. Bootstrap resampling was subsequently used to evaluate the stability of this partition, and pattern scores were compared among groups using the Kruskal–Wallis test. Hierarchical clustering analyses, silhouette score calculations, and subsequent statistical analyses were performed using R software (version 4.0.2, R Statistics, Vienna, Austria) and visualized with Orange software (version 3.36.1, Bioinformatics Laboratory, University of Ljubljana, Ljubljana, Slovenia).

## 3. Results

### 3.1. Baseline characteristics

We included 423 patients (175 men and 248 women) with a mean age of 50.3 years. Patients with low back pain (n = 146, 34.5%), migraine (n = 38, 9.0%), irritable bowel syndrome (n = 92, 21.7%), ankle sprain (n = 71, 16.8%), knee pain (n = 54, 12.8%), carpal tunnel syndrome (n = 18, 4.3%), and dysmenorrhea (n = 4, 0.9%) were included. The mean duration of symptoms was 24.7 months, with a mean pain intensity of 5.8 points on a 0 to 10 numerical rating scale.

### 3.2. Acupoint combinations for pain control

**Figure 2.**
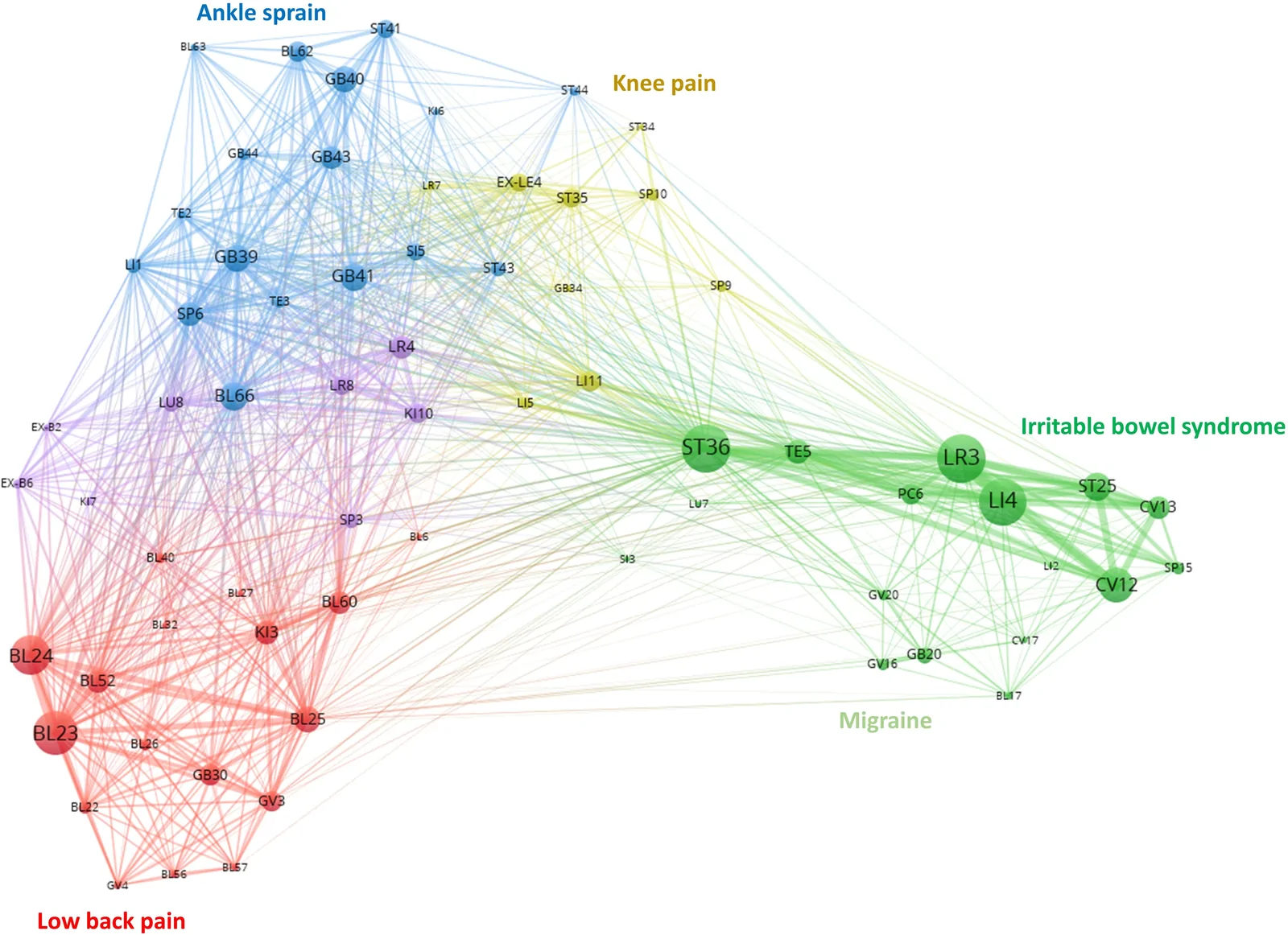
Acupoint network for pain management. Of the 177 acupoints, 68 were used >10 times in pain management. The most frequently used acupoints for pain management were ST36 (n = 113, degree = 66), LR3 (n = 112, degree = 41), and LI4 (n = 109, degree = 40). The size of the nodes represents the frequency of acupoint use, and the thickness of the links indicates the co-occurrence frequency.

Of the 177 acupoints, 68 were used >10 times in pain management. The most frequently used acupoints were ST36 (n = 113, degree = 66), LR3 (n = 112, degree = 41), and LI4 (n = 109, degree = 40). The most common acupoint combinations for pain control were LI4-LR3 (n = 94), BL23-BL24 (n = 83), LI4-CV12 (n = 71), LR3-CV12 (n = 67), LI4-ST25 (n = 57), LI4-ST36 (n = 56), and LR3-ST36 (n = 56) (Fig. 2).

The primary acupoints in the upper and lower limbs (ST36, LI4, and LR3) were used across various pain disorders. Conversely, certain local acupoints exhibited more condition-specific applications. For instance, BL23 and BL24 were primarily used for the treatment of low back pain, whereas GB40 and BL62 were used for the management of ankle sprains.

### 3.3. Pattern-related profiles of acupoint use

Mean pattern scores were calculated for the 30 most frequently prescribed acupoints. For SP6, the mean scores were –0.9 for deficiency–excess, –0.5 for cold–heat, and 0.9 for dryness–dampness, corresponding to relatively greater deficiency, cold, and dampness among patients for whom it was prescribed. Table 1 presents the corresponding mean pattern scores for each acupoint. The mean scores across the 30 acupoints were –0.12, –0.09, and 0.48 for the deficiency–excess, cold–heat, and dryness–dampness patterns, respectively, whereas the corresponding mean scores across all cases were 0.09, –0.07, and 0.48.

**Table 1:** Mean pattern scores of the 30 most frequently prescribed acupoints for pain management.

Acupoints	Deficiency–Excess	Cold–Heat	Dryness–Dampness
ST36	0.08	−0.04	0.41
LR3	−0.13	−0.28	0.02
LI4	−0.23	−0.43	0.08
BL23	−0.15	0.01	0.31
BL24	−0.38	−0.02	0.35
CV12	−0.34	−0.51	0.15
ST25	−0.21	−0.32	−0.08
BL66	−0.89	−0.37	0.69
GB39	−0.77	−0.45	0.68
GB41	0.02	−0.12	0.88
BL25	−0.28	−0.09	0.41
GB40	0.36	0.41	0.73
TE5	−0.13	−0.32	−0.17
SP6	−0.91	−0.53	0.87
KI3	0.58	0.62	0.37
CV13	−0.34	−0.52	0.10
BL52	−0.12	−0.04	0.59
BL60	0.80	0.59	0.37
LR4	−0.35	−0.29	0.35
GB43	−0.38	−0.10	0.67
GB30	0.62	−0.09	0.98
GV3	1.24	0.02	0.87
LI11	0.52	0.16	0.66
PC6	−0.26	−0.65	−0.07
BL62	0.42	0.58	0.56
KI10	−0.49	0.12	0.66
LR8	−0.74	−0.21	0.67
ST41	−0.18	0.29	0.58
SI5	0.17	0.23	1.09
LU8	−1.09	−0.50	0.50

**Figure 3.**
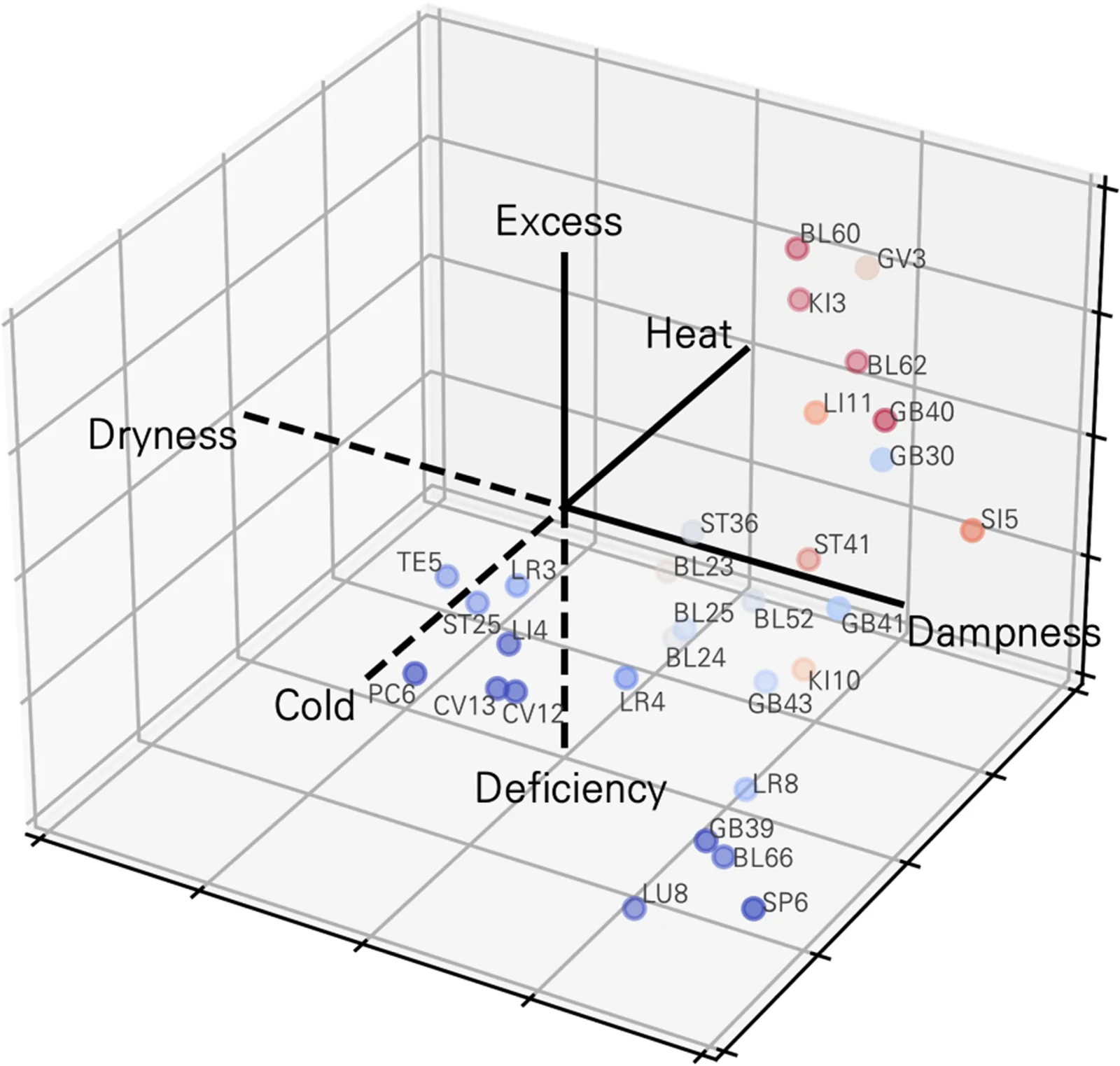
Three-dimensional visualization of acupoint-associated pattern profiles. Mean pattern scores for each acupoint were plotted along deficiency–excess, cold–heat, and dryness–dampness dimensions. Solid lines indicate values toward excess, heat, and dampness, whereas dotted lines indicate values toward deficiency, cold, and dryness. The color gradient represents the cold–heat dimension, with blue indicating colder and red indicating hotter profiles.

These scores were visualized in a three-dimensional space, with axes representing deficiency–excess, cold–heat, and dryness–dampness (Fig. 3). Acupoints with similar pattern profiles were located close together. For instance, acupoints in the lower right quadrant with a bluish color, such as LU8 and SP6, were located in the region corresponding to relatively greater deficiency, cold, and dampness.

### 3.4. Clustering acupoints based on pattern-profile similarity

Silhouette scores for two- to six-cluster solutions were 0.45, 0.42, 0.44, 0.43, and 0.43, respectively. Thus, silhouette analysis did not identify a clearly dominant cluster solution, and the highest value was observed for the two-cluster solution. We retained the five-cluster partition as a higher-resolution representation of the heterogeneity in pattern scores without implying an optimal or definitive classification. Bootstrap resampling yielded Jaccard similarity coefficients of 0.88, 0.99, 0.96, 0.82, and 0.68 for the 5 groups. The Kruskal–Wallis test showed differences in all 3 pattern scores among these groups (all *P* < .001). These analyses support the stability of the selected partition but do not establish 5 clusters as the uniquely optimal classification.

**Figure 4.**
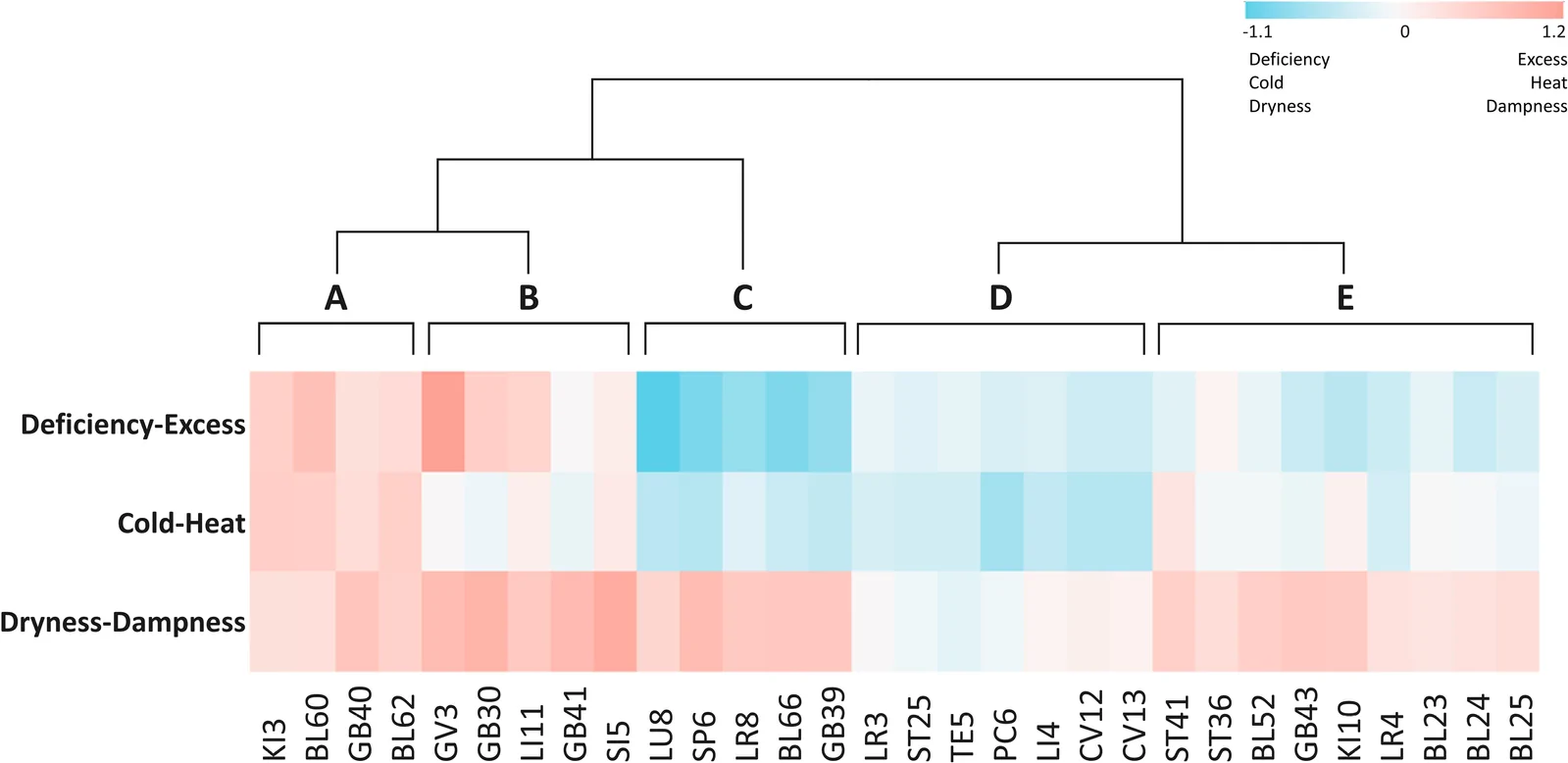
Hierarchical clustering of acupoint-associated pattern profiles. The dendrogram and heatmap illustrate an exploratory five-group partition based on mean deficiency–excess, cold–heat, and dryness–dampness scores. The 5 groups represent relative similarities in clinician-assigned pattern profiles and should not be interpreted as a definitive clinical classification.

The 5 groups included (Fig. 4): KI3, BL60, GB40, and BL62 (A); GV3, GB30, LI11, GB41, and SI5 (B); LU8, SP6, LR8, BL66, and GB39 (C); LR3, ST25, TE5, PC6, LI4, CV12, and CV13 (D); and ST41, ST36, BL52, GB43, KI10, LR4, BL23, BL24, and BL25 (E) (Table 2).

**Table 2:** Pattern scores of the 5 exploratory acupoint groups.

Groups	Deficiency–Excess	Cold–Heat	Dryness–Dampness
A	0.54 ± 0.20	0.55 ± 0.09	0.51 ± 0.18
B	0.51 ± 0.48	0.04 ± 0.15	0.89 ± 0.16
C	−0.88 ± 0.14	−0.41 ± 0.13	0.68 ± 0.13
D	−0.23 ± 0.09	−0.43 ± 0.14	0.00 ± 0.12
E	−0.25 ± 0.17	−0.01 ± 0.16	0.48 ± 0.14

Values are presented as mean ± standard deviation (SD).

## 4. Discussion

Using real-world clinical data, we analyzed commonly prescribed acupoints for pain management and their relationships with deficiency–excess, cold–heat, and dryness–dampness scores. The analysis revealed variation in clinician-assigned pattern scores across commonly used acupoints. Hierarchical clustering provided a descriptive representation of similarities among these patterns.

In clinical practice, ST36, LR3, and LI4 were the most commonly used acupoints for pain management, consistent with prior studies.^[5,6]^ These acupoints have also been frequently studied in relation to acupuncture analgesia, including descending pain modulation and central regulation.^[5]^ They were applied to a diverse range of painful conditions, whereas other acupoints, such as BL23 and GB40, were specifically targeted for certain types of pain. These findings emphasize the principles of commonality and specificity in acupoint selection.^[17]^ Although major acupoints have broad applications, they may show less therapeutic specificity. In contrast, acupoints with narrower indications may allow more targeted treatment.

Pattern scores differed across acupoints in this dataset. For example, GV3 was prescribed to patients with relatively greater excess scores, whereas LU8 was more often used in those with relatively greater deficiency scores. These findings suggest heterogeneity in the pattern-related contexts in which acupoints are used, even among patients treated for pain. However, these associations should not be interpreted as intrinsic or validated pattern properties of individual acupoints. Although acupuncture treatment at specific acupoints has shown clinical efficacy across various conditions,^[18,19]^ pattern-based acupuncture has been suggested to promote broader whole-body improvement.^[20]^ Acupuncture patterns are classified based on meridians (lung and large intestine meridians), organ systems (liver qi stagnation and spleen deficiency), principles (yang/yin and heat/cold), and body constituents (qi, blood, fluid, and essence).^[21]^ Spatial relationships between acupoint indications and body locations have been reported in studies of meridian-based patterns.^[22]^ Furthermore, associations between acupoint selection and organ-based patterns have been examined in internal disorders, such as functional gastrointestinal disorders.^[7,23]^ While principle-based patterns are widely used in clinical practice,^[24]^ their relationship with acupoint selection remains uncertain. Therefore, we examined the balance between opposing patterns to provide a broader view of the pattern-related context of acupoint use.

The exploratory clustering grouped acupoints with similar clinician-assigned pattern profiles. This data-driven approach may help summarize complex prescription patterns, but the five-group solution should not be regarded as a discrete clinical classification. In particular, the modest and similar silhouette scores across candidate solutions indicate that the underlying structure may be gradual rather than composed of clearly separated categories. Thus, the clusters are best interpreted as a descriptive representation of relative similarities among commonly used acupoints. Importantly, the observed pattern–acupoint associations cannot be interpreted independently of disease-related and clinician-related prescribing factors.

Acupoint selection may reflect disease or pain location, pattern assessment, clinical preference, or a combination of these factors. For example, ankle sprain may be associated both with particular clinical characteristics and with the use of local acupoints around the ankle. This makes it difficult to distinguish disease-specific selection from pattern-related selection. Similarly, the predominance of certain disorders, particularly low back pain, may have influenced the profiles of frequently prescribed acupoints. Clinic- and clinician-specific prescribing preferences may also have contributed to the observed relationships. Thus, the independent contribution of pattern identification to acupoint selection cannot be determined from the present data.

Our study has several limitations. First, the number of patients across different pain conditions was unevenly distributed. Approximately 30% of patients presented with low back pain, while other disorders, such as dysmenorrhea, were less common. This may be attributed to the predominance of musculoskeletal problems in outpatient clinics.^[25]^ However, we included a diverse range of pain conditions, including musculoskeletal disorders, irritable bowel syndrome, and migraine. Second, because the study population was limited to patients with pain, the observed pattern profiles may partly reflect the pattern distribution of this population. For example, relatively high dampness scores were observed both across frequently prescribed acupoints and in the overall sample. Therefore, these findings may not generalize to acupoint use in non-pain conditions. Third, although we included deficiency–excess, cold–heat, and dryness–dampness patterns, we did not consider yin-yang and exterior–interior patterns among the 8 principal-based patterns. Yin-yang patterns are more difficult to assess independently because they broadly integrate several other pattern characteristics. Moreover, we did not assess exterior–interior patterns because the study focused on patients with pain. Exterior-interior patterns are more commonly used to distinguish superficial conditions, such as the common cold, from internal conditions involving abdominal or other internal symptoms.^[26]^ Fourth, the pattern scores were clinician-assigned semi-quantitative ratings rather than standardized diagnostic measurements. Formal diagnostic criteria, clinician calibration, and inter-rater reliability assessment were not available because each patient was evaluated by a single clinician. Fifth, disease type and clinic- or clinician-specific prescribing patterns may have confounded the observed associations. The present data do not allow these influences to be disentangled from the contribution of pattern identification to acupoint selection. Finally, as the data were collected from Korean Medicine clinics within a single healthcare and cultural context, the identified pattern–acupoint relationships may not generalize to other TEAM systems or non-Korean settings.

Our findings provide an exploratory description of how clinician-assigned pattern assessments vary across commonly used acupoints in real-world pain practice. These findings suggest that clinical acupoint selection may reflect the combined influence of disease- and symptom-based indications and broader pattern assessments. Future prospective studies using standardized pattern assessments and adjustment for disease- and clinician-related factors are needed to determine the independent contribution of pattern identification to acupoint selection.

## Author contributions

**Conceptualization:** Da-Eun Yoon, Yoonjeong Seo, Younbyoung Chae.

**Data curation:** Shin Ho Kong, Changwoo Nam, Joowon Hwang, Karam Kim, Man-Heum Kwon, Hyun-Woo Jin.

**Formal analysis:** Da-Eun Yoon, Younbyoung Chae.

**Funding acquisition:** Yeonhee Ryu.

**Investigation:** Younbyoung Chae.

**Methodology:** Yoonjeong Seo.

**Project administration:** Yeonhee Ryu, Younbyoung Chae.

**Supervision:** In-Seon Lee.

**Validation:** Heeyoung Moon.

**Writing – original draft:** Da-Eun Yoon, Younbyoung Chae.

**Writing – review & editing:** Yoonjeong Seo, Heeyoung Moon, Yeonhee Ryu, In-Seon Lee.

## References

[1] Birch S Alraek T. Traditional East Asian Medicine: how to understand and approach diagnostic findings and patterns in a modern scientific framework? Chin J Integr Med. 2014;20:336–40. 10.1007/s11655-014-1809-3 24788086

[2] Lee YS Chae Y. Pattern Identification and acupuncture prescriptions based on real-world data using artificial intelligence. East Asian Sci Technol Soc. 2025;19:267–84.

[3] Hwang YC Lee YS Ryu Y Lee IS Chae Y. Statistical inference of acupoint specificity: forward and reverse inference. Integr Med Res. 2020;9:17–20. 10.1016/j.imr.2020.01.005 PMC7078453 32195113

[4] Lee YS Ryu Y Chae Y. Acupoint selection based on pattern identification results or disease state. Integr Med Res. 2020;9:100405. 10.1016/j.imr.2020.100405 PMC7176939 32337153

[5] Lee IS Chae Y. Identification of major traditional acupuncture points for pain control using network analysis. Acupunct Med. 2021;39:553–4. 10.1177/0964528420971309 33319596

[6] Hwang YC Lee IS Ryu Y Lee MS Chae Y. Exploring traditional acupuncture point selection patterns for pain control: data mining of randomised controlled clinical trials. Acupunct Med. 2020;39:184–91. 10.1177/0964528420926173 32567332

[7] Moon H Ryu Y Lee IS Chae Y. Acupuncture treatment for functional gastrointestinal disorders: identification of major acupoints using network analysis. Integr Med Res. 2023;12:100970. 10.1016/j.imr.2023.100970 PMC10407428 37559923

[8] Ho LT Chung VC Wong CH. Evaluating traditional Chinese medicine diagnostic instruments for functional dyspepsia: systematic review on measurement properties. Integr Med Res. 2021;10:100713. 10.1016/j.imr.2020.100713 PMC7903347 33665098

[9] Jung WM Park IS Lee YS. Characterization of hidden rules linking symptoms and selection of acupoint using an artificial neural network model. Front Med. 2019;13:112–20. 10.1007/s11684-017-0582-z 29651775

[10] Park M Kim MH Park SY Choi I Kim C-E. Individualized diagnosis and prescription in traditional medicine: decision-making process analysis and machine learning-based analysis tool development. Am J Chin Med. 2022;50:1827–44. 10.1142/S0192415X2250077X 36056467

[11] Kim CH Yoon DE Lee YS. Revealing associations between diagnosis patterns and acupoint prescriptions using medical data extracted from case reports. J Clin Med. 2019;8:1663. 10.3390/jcm8101663 PMC6832135 31614636

[12] Waltman L van Eck NJ Noyons ECM. A unified approach to mapping and clustering of bibliometric networks. J Inform. 2010;4:629–35.

[13] World Health Organization Regional Office for the Western Pacific. WHO International Standard Terminologies on Traditional Medicine in the Western Pacific Region. WHO Regional Office for the Western Pacific; 2007.

[14] Nielsen F. Hierarchical Clustering. Introduction to HPC with MPI for Data Science: Springer, Cham; 2016.

[15] Ward JH Jr Hierarchical grouping to optimize an objective function. J Am Stat Assoc. 1963;58:236–44.

[16] Rousseeuw PJ. Silhouettes: a graphical aid to the interpretation and validation of cluster analysis. J Comput Appl Math. 1987;20:53–65.

[17] Lee YS Ryu Y Yoon DE. Commonality and specificity of acupuncture point selections. Evid Based Complement Alternat Med. 2020;2020:2948292. 10.1155/2020/2948292 PMC7403905 32802119

[18] Birch S Hesselink JK Jonkman FA Hekker TAM Bos A. Clinical research on acupuncture. Part 1. What have reviews of the efficacy and safety of acupuncture told us so far? J Altern Complement Med. 2004;10:468–80. 10.1089/1075553041323894 15253851

[19] Kelly RB Willis J. Acupuncture for pain. Am Fam Physician. 2019;100:89–96. 31305037

[20] Birch S. Treating the patient not the symptoms: acupuncture to improve overall health – evidence, acceptance and strategies. Integr Med Res. 2019;8:33–41. 10.1016/j.imr.2018.07.005 PMC6428918 30949430

[21] World Health Organization. International Classification of Diseases for Mortality and Morbidity Statistics (11th Revision). World Health Organization; 2019.

[22] Jung WM Lee T Lee IS. Spatial patterns of the indications of acupoints using data mining in classic medical text: a possible visualization of the meridian system. Evid Based Complement Alternat Med. 2015;2015:457071. 10.1155/2015/457071 PMC4619912 26539224

[23] Kim SY Hong SH Park JW. Analysis of diagnostic decision in acupuncture from the actual functional dyspepsia patient’s clinical information. Integr Med Res. 2020;9:100419. 10.1016/j.imr.2020.100419 PMC7236055 32455110

[24] Bae KH Lee Y Park KH Yoon Y Mun S Lee S. Perception of cold and heat pattern identification in diseases: a survey of Korean medicine doctors. Integr Med Res. 2017;6:26–32. 10.1016/j.imr.2016.10.004 PMC5395675 28462141

[25] Oh IH Yoon SJ Park M An SH. Disease-specific differences in the use of traditional Korean medicine in Korea. BMC Complement Altern Med. 2015;15:141. 10.1186/s12906-015-0657-9 PMC4440311 25935842

[26] Jiao Y Liu J Jiang L. Guidelines on common cold for traditional Chinese medicine based on pattern differentiation. J Tradit Chin Med. 2013;33:417–22. 10.1016/S0254-6272(13)60141-7 PMC7148786 24187858

